# Exploring Malaysian parents' and teachers' cultural conceptualization of adolescent social and emotional competencies: A qualitative formative study

**DOI:** 10.3389/fpubh.2023.992863

**Published:** 2023-03-23

**Authors:** Nur Hazwani Abd Hadi, Marhani Midin, Seng Fah Tong, Lai Fong Chan, Hajar Mohd Salleh Sahimi, Abdul Rahman Ahmad Badayai, Norsinar Adilun

**Affiliations:** ^1^Department of Psychiatry, Faculty of Medicine, National University of Malaysia, Kuala Lumpur, Malaysia; ^2^Department of Family Medicine, Faculty of Medicine, National University of Malaysia, Kuala Lumpur, Malaysia; ^3^Centre for Research in Psychology and Human Well-Being (PsiTra), Faculty of Social Sciences and Humanities, National University of Malaysia, Kuala Lumpur, Malaysia

**Keywords:** social-emotional learning, cultural conceptualization, adolescents, Malaysia, emotional suppression

## Abstract

**Introduction:**

Global implementation of social and emotional learning (SEL) has been suggested to incorporate a systematic cultural adaptation process which relies on ground-up empirical data of a target cultural group in tailoring a culturally sensitive SEL intervention. Preliminary formative studies among local parents and educators were done to explore the conceptualization of social and emotional competencies (SECs) in various cultural settings, such as the continent of Africa and among the indigenous and refugee groups. Unfortunately, little scholarship has been devoted to studying the SEL adaptation process in Southeast Asian regions. This formative study aimed to explore Malaysian parents' and teachers' cultural conceptualization of adolescent SECs.

**Methods:**

This qualitative study interviewed 12 Malaysian parents and 10 Malaysian teachers comprising of Malay (82%), Chinese (9%) and Indian (9%) races in an online focus group discussion. Sampling is purposive to parents of adolescents and teachers at secondary school only. Data were analyzed thematically to determine the culturally sensitive SEL constructs for Malaysian adolescents.

**Results:**

All themes and sub-themes of SEC regarded as crucial for Malaysian adolescents are aligned with CASEL's five domains of competencies. Our findings extended the conceptualization of subskills under CASEL's relationship skills and responsible decision-making domains, which reflect Asian cultural values. The main themes of social competency: (a) preserving interpersonal relationships, (b) utilizing intrapersonal skills, and (c) communicating effectively, are shared with the established CASEL constructs. However, the underlying subthemes denote the unique cultural manifestation of social competency in Malaysia. Two of the emotional competency themes represent the established CASEL constructs: (a) practicing self-regulation, (b) demonstrating help-seeking behavior, and the other two themes signify Asian values: (c) upholding altruism, and (d) maintaining cultural display rules.

**Discussions:**

This formative study revealed the habitual use of experiential and expressive suppressions as adaptive emotion regulation strategies in Malaysian collectivist culture and offered a potential alternative emotion regulation pathway suitable for Malaysian adolescents. It also informed the feasibility of implementing SEL modules developed based on the CASEL framework in Malaysia and suggested two key lessons to enhance the cultural sensitivity of SEL in Malaysia: effective, respectful communication and expressive writing.

## 1. Introduction

Social and emotional learning (SEL) is an umbrella term referring to a systematic process of educating intrapersonal, interpersonal, and cognitive competencies that help students navigate their feelings, thoughts, and actions to thrive in school, work, and life (1, 2). Research has shown the effectiveness of kindred approaches of SEL in improving students' academic outcomes, positive wellbeing, and prosocial behaviors (3–5). Due to its effectiveness in nurturing holistic students through healthy social and emotional development, SEL has become the zeitgeist of current education. Unfortunately, SEL constructs are foundationally developed in Western, Educated, Industrialized, Rich, and Democratic (WEIRD) contexts (6, 7). Emerging research stresses the need to adapt SEL according to sociocultural background due to the evidence that shows differential effects of SEL intervention on children based on sociocultural factors. For instance, SEL curriculums aimed to reduce internalizing problems that have been found to be effective with children from a particular cultural background but not with others (8).

The global implementation of SEL across countries with different cultural backgrounds presents two concerning issues. First, SEL intervention research begins with the aim of documenting efficacy for broader and diverse populations without focusing on the fundamental process of tailoring the intervention to specific cultural needs (8, 9). SEL adaptation research has been suggested to move beyond descriptive analyses based on the demographic distribution of participants. Merely including participants from broader cultural groups does not necessarily imply the adaptability of the intervention to a particular cultural population. Second, most available research on SEL adaptation only reported surface-level modifications to the program materials, which involve language translation, duration, visual aids, and examples relevant to a particular cultural context (10).

A relatively new direction in improving the effectiveness of SEL intervention for diverse cultural populations is to rely on ground-up empirical data specific to the target cultural group. This involves establishing formative research to explore the target population's cultural beliefs, norms, and values, which will inform the weight and the nature of modifications needed to tailor a culturally sensitive SEL intervention. SEL implementation in other countries should move toward a systematic cultural adaptation process, whereby studying the cultural beliefs and values comes first before implementing SEL intervention and further generalizing its adaptability to a particular cultural context (8, 11). Cultural adaptation is defined as “a systematic and thoughtful process of incorporating culture into evidence-based interventions” (12). This practice has been found to increase the effectiveness of intervention among the culturally distinct population by grounding the intervention practices in the lived experience of the target community (13). To date, various frameworks of cultural adaptation have been established as guidance to ensure the fidelity of intervention while insuring optimal cultural appropriateness (14–16). In each model, the process of cultural adaption is fundamentally started from the ground up by gathering information about the target community and assessing the community's cultural beliefs, norms, and values (13, 17).

In the burgeoning effort to culturally adapt SEL intervention from the ground up, researchers have studied the conceptualizations of social and emotional competencies (SECs) from the perspectives of local caregivers and educators (18–21). This emic approach in exploring the cultural beliefs, norms, and values pertaining to SEC is crucial before implementing SEL in a non-WEIRD context. According to Bronfenbrenner's Ecological System theory, home and school comprise the microsystem that directly influences children's social and emotional development (22). In this sense, parents' and teachers' conscious and unconscious beliefs regarding important domains of SEC represent the cultural model that shapes children's development through specific parenting and teaching practices, emotional socialization, and values being imposed (23). Studying the cultural model that governs the conceptualization of SEC in a particular context discards assumptions about the valuable skills needed for children and students to succeed in a particular culture.

In order to implement SEL effectively in a new cultural context, the process of cultural adaptation is essential because the gist of SEL, which is to nurture SECs, is significantly influenced by cultural norms and values. Matsumoto et al. proposed that different cultural practices demonstrate different common strategies to manage and regulate emotions. Collectivist countries with a cultural orientation of social embeddedness, harmony, and hierarchy tend to use suppression more, whereas individualist countries that valued autonomy and egalitarianism tend to use suppression less and reappraisal more (24, 25). Another study suggested that the consequences of emotional suppression are culture-specific. Americans holding Western-Europe individualist values experienced more negative emotions due to habitual suppression compared to those with Asian collectivist values (26). Consistent results found in a cross-country study assessing the effect of emotional suppression to life satisfaction among American and Hong Kong Chinese. American participants reported lower satisfaction in the high-suppression condition, but no difference was found between the high-suppression and the control condition among the Chinese. This indicates that the use of emotional suppression only undermines the life satisfaction of those who hold individualist values and not collectivist ones (27). A study of emotion regulation (ER) among pre-schoolers found that American children are more expressive of their negative emotions in the disappointing gift paradigm compared to the Chinese and Japanese children who display neutral response through expressive suppression. This may be due to the different socialization of emotional expression across cultures (28). Therefore, individuals' cultures and values are known to impose norms regarding ER styles (29) and thus influence the understanding and belief toward elements of SECs. Hence, this is why SEL practice warrants cultural adaptation when being implemented in other culturally distinct populations.

Currently, the ground-up approach in SEL adaptation research among the non-WEIRD contexts has studied the refugees (18) and indigenous societies (19), conflict-affected regions (20), and the continent of Africa (21). Unfortunately, formative research to inform the cultural adaptation of SEL in Southeast Asian countries is still lacking. To date, Singapore is the only country that has systematically studied SEL constructs from the ground up and documented an SEL framework that is adapted to Asian collectivist culture (30, 31). However, Singapore itself is an educated, industrialized, rich, and democratic country, except it is not Western. SEL adaptation research from Singapore is not sufficient in representing the non-WEIRD context of the Southeast Asia regions. A country like Malaysia represents the non-WEIRD context for its developing, middle-income state and offers a unique setting due to its collectivist and multicultural population. In Malaysia, educating holistic students has long been the core aspiration of the National Education Philosophy (32). Unfortunately, this aspiration has yet to be reflected in the current state of education. As a developing country, the Malaysian Education Blueprint mainly focuses on preparing globally competitive students by empowering the science, technology, engineering, and mathematics (STEM) academic curriculums, resulting in academic- and exam-oriented students (33). This quality alone is insufficient to ensure Malaysian students thrive in the challenges of the current volatility, uncertainty, complexity, and ambiguity (VUCA) world (34, 35).

This current study pursues a research question of what are Malaysian parents' and teachers' cultural conceptualization of adolescent SECs? This formative research addresses the scarcity of SEL adaptation research in the Southeast Asian regions representing a non-WEIRD context. Malaysian parents and teachers were interviewed to investigate their beliefs regarding crucial domains and skills for students to be considered socially and emotionally competent in Malaysia. The findings were thematically analyzed to determine what skills are valued and endorsed by Malaysian culture as necessary for students' success. In order to inform theory and practices of further efforts in SEL adaptation in Malaysia, we compare our findings of culturally sensitive SEL constructs with the existing, well-established Collaborative for Academic, Social, and Emotional Learning (CASEL) framework (3, 36).

## 2. Method

### 2.1. Study context

This study is part of an extensive research project on implementing and adapting a culturally sensitive SEL framework for Malaysian adolescents in secondary schools. While many WEIRD countries begin SEL curriculums early and continue through higher education, we have two critical rationales for focusing on Malaysian adolescents only as a start. First, due to its exploratory phase, the adolescent is the ideal age group from which we can gather qualitative formative insight to better inform our framework development that suits the Malaysian school culture and students' needs. Second, the rising mental health disorder among Malaysian adolescents needs to be urgently addressed by nurturing their SECs to enhance protective factors in navigating life stressors.

This study is part of a bigger research on developing a Malaysian SEL framework. In this research, our research group conducted two formative studies simultaneously: the adolescent study, and the parents and teachers study (this current study). While the adolescent study explored Malaysian adolescents' social and emotional needs, this study explored Malaysian parents' and teachers' perspectives on what constitutes crucial SECs for adolescents to succeed holistically. The scope of this current study was to explore the conceptualization of SEC from Malaysian cultural perspectives as a ground-up approach to determine the culturally sensitive SEL constructs for Malaysian adolescents.

### 2.2. Procedure

A poster detailing our inclusion criteria for participants was produced, along with some brief information about the topic and medium of discussion. The poster was distributed through WhatsApp and Telegram groups, social media platforms, emails, and word-of-mouth by all the researchers involved. In addition, we engaged with some public schools in Malaysia to recruit secondary school teachers. Participants registered their interest by filling up the online registration Google form provided through the QR code on the poster. In the online form, participants were instructed to read the research information sheet and fill up the consent form survey before they were able to submit their online registration. Registered participants were then contacted to set an appointment for their online focus group session. Reminders and Zoom links were sent to participants a day before the appointment to improve efficiency.

In each session, participants were briefed regarding the research topic, consent, and online focus group rules and technicalities. The ground rules include respecting everyone's opinions and speaking one at a time, and the confidentiality of participants' identity and data was reserved only for analysis purpose by the research team. Participants were informed about the focus of the discussion, which is to explore what constitutes important SEC for Malaysian adolescents to succeed holistically. However, they were not briefed in detail on SEL specifically before the interview. Each focus group discussion was video recorded with consent from all participants. Groups that had only Malay participants were interviewed in Malay, while groups that had Chinese or Indian participants agreed to be interviewed in English as per request by participants. Only researchers of this research have the accessibility to participants' data for analysis purposes. All participants were given the honorarium of RM100 for their time and input shared in the interview. Ethical approval to conduct this research was obtained from the Research Ethics Committee of the National University of Malaysia on 20 April 2021. Approval to interview the secondary school teachers was obtained from the Malaysian Educational Research Information System (ERAS) on 2 August 2021.

### 2.3. Participants

The sampling of participants was purposive to some inclusion criteria: (i) participants are parents who have at least one adolescent child in secondary schools or teachers who teach secondary school students only and (ii) participants must incorporate all groups of socioeconomic status in Malaysia, and the total household income classified as B40 (RM4,851 and below), M40 (RM4,851–RM10,970), and T20 (RM10,970 and above). This is a standardized income classification that has been used formally in Malaysia (37). After a few focus group discussion sessions involving predominantly Malay parents and teachers, we added another inclusion criterion: (iii) participants must incorporate other races in Malaysia, such as Chinese and Indian, to better inform our analysis. Since parents and teachers are more informed about adolescents' social and emotional needs as compared to adults without adolescent children, the latter group was not involved in the sample. The descriptive statistics of the participants are summarized in Table 1.

**Table 1:** Descriptive statistics of participants.

	***n***	**%**
Parents	12	54.5
Teachers	10	45.5
**Parents' SES**		
B40	3	25.0
M40	6	50.0
T20	3	25.0
**Teachers' SES**		
B40	2	20.0
M40	5	50.0
T20	3	30.0
**Parents' gender**		
Female	8	66.7
Male	4	33.3
**Teachers' gender**		
Female	7	70.0
Male	3	30.0
**Parents' race**		
Malay	12	100.0
Chinese	-	
Indian	-	
**Teachers' race**		
Malay	6	60.0
Chinese	2	20.0
Indian	2	20.0

A total of 15 parents and 18 teachers registered their interest to participate in this research through the Google form; however, only 12 parents and 10 teachers were interviewed in the online focus groups due to unreachable contact numbers or participants' unavailability to attend the focus group appointment. Two of the unavailable teachers withdrew on the day of the focus group appointment without prior notice; hence, the focus group was left with three instead of four participants. We purposely chose to conduct mini focus groups involving three to four participants in a group. We had three focus groups among the parents and three focus groups among the teachers. Mini-focus groups were considered ideal with the online setting of our focus group discussion. The nature of virtual meetings suffers the drawback of online/Zoom fatigue (38). With mini-focus groups, the duration of the whole session was maintained below 2 h, approximately around 100 min. Moreover, in that optimum time, we were able to gain in-depth and detailed insight from each of our small number of participants. We grouped parents and teachers separately to maximize the discussion dynamic focusing on specific home and school settings. The participants were grouped based on their available dates to attend the focus group session.

### 2.4. Data collection

Our focus group discussions were done online through Zoom meetings due to the COVID-19 pandemic movement restriction order in Malaysia. Based on our experience, the online interview is an effective method to gather qualitative data since participants felt more comfortable sharing their input in the comfort of their homes without the added pressure of being in a foreign setting of a face-to-face group discussion with new people. This supports existing findings that suggest online interviews and promote greater engagement from research participants (39). The interview was moderated by the first author and supported by another experienced researcher to ensure the discussions were aligned with the research aim. The whole process of data collection lasted for 8 months, from June 2021 to January 2022.

The interview question guide was designed by the moderator with the supervision of other researchers. The interview guide was first tested with the researcher's family members and acquaintances who fit the research inclusion criteria to inform further improvisation process. Due to the unfamiliar concept of SEC among Malaysian parents and teachers, questions addressing social and emotional qualities need to be asked separately to allow deeper introspection and retrospection of both interpersonal and intrapersonal skills valued within the Malaysian cultural context. Parents and teachers were asked about “*what qualities a socially competent student/child would have?,” “what qualities an emotionally competent student/child would have?,”* and “*what skills are needed for a student/child to be successful holistically (mentally, physically, spiritually)?.”* These open-ended questions allow for the exploration of conceptual meanings of SECs according to Malaysian norms and cultures. Follow-up questions were asked for participants to expand their answers with specific examples and contexts where the qualities were portrayed by their students/children. The co-construction of meanings was enhanced by asking other participants whether they agreed or disagreed with the qualities articulated to add more nuances toward particular answers.

### 2.5. Data analysis

The focus group discussions were transcribed verbatim by research assistants and checked for accuracy by the first author. In the transcription, participants were relabeled according to their relationship with adolescents (parents or teachers) and a number (e.g., Parent #1). Malay verbatim quoted in this study was translated to English for reporting purposes. Language translation accuracy was cross-checked by another first-language Malay-speaking and second-language English-speaking research assistant.

Data analysis was aided by using NVivo (released in March 2020) as data management software. Our analysis was entirely inductive due to the exploratory nature of this current study. The inductive thematic analysis was guided by Braun and Clarke's six-step approach for reflexive thematic analysis, which involves (i) familiarization of the interview transcript, (ii) coding of data, (iii) generating themes, (iv) reviewing themes, (v) defining and naming themes, and (vi) writing up a report (40). The findings of SEC were separately analyzed. The first author coded data with frequent discussion with other researchers as peer review for interpretation of the text. The coding was guided by the research aim and question and subsequently categorized into themes and subthemes. The process of categorizing was reviewed during discussions to organize findings into different but connected abstract levels between themes and subthemes.

We found remarkable similarities between parents and teachers in their perspectives on SECs; thus, they were combined during the analysis. No specific theme emerged that solely represents a particular gender, SES, or race, indicating that the conceptualization of SEC in the Malaysian context was uniformly shared despite demographic background. This study utilized data saturation points to determine the sample size of participants, which is a standard approach for qualitative research (41). Data saturation was achieved when there was no longer new additional finding or theme identified in our analysis. The themes and subthemes were discovered and saturated after the third parents' focus groups and second teachers' focus groups. We added another focus group consisting of non-Malay teachers to confirm our analyses and themes among other races in Malaysia, making altogether 12 parents and 10 teachers interviewed. The themes and subthemes discovered from our inductive thematic analysis were then compared with the established CASEL framework to provide insight into how similar or distinct the Malaysian conceptualization of SEC is from the CASEL constructs.

## 3. Result

Malaysian parents' and teachers' views of SEC are not entirely distinct from the established CASEL framework. All themes and subthemes of SEC regarded as crucial for Malaysian adolescents are aligned with CASEL's five core domains of competencies, namely, self-awareness, self-management, social awareness, relationship skills, and responsible decision-making. As in the CASEL framework, there are specific subskills that made up the competency for each domain. Our findings indicate the presence of unique cultural manifestations of subskills under the domain of relationship skills and responsible decision-making.

Each of our themes/subthemes is found to match with one of the five CASEL competency domains according to the established definition of the domains. Some of the themes/subthemes reflect the standard subskills under CASEL domains, while others are additional subskills that reflect Asian cultural values. These additional subskills extended the conceptualization of CASEL domains of competencies that are unique to Malaysian culture. **Table 4** categorizes the themes and subthemes of SEC according to their parallel definition of CASEL domains and differentiates findings that belong to standard CASEL subskills with the extended subskills related to Asian values. The formal definition of each CASEL domain is included as a reference (36).

Our analyses of social competency yielded the following three overarching themes: (a) preserving interpersonal relationships, (b) utilizing intrapersonal skills, and (c) communicating effectively. Some underlying subthemes denote different displays of Asian cultural manifestations of social competency. Table 2 summarizes the main themes and subthemes of social competency based on Malaysian parents' and teachers' perspectives. Emotional competency can be described with four overarching themes. Two of the themes are aligned with the CASEL subskills: (a) practicing self-regulation and (b) demonstrating help-seeking behavior, while the other two themes are rooted in Asian values: (c) upholding altruism and (d) maintaining cultural display rules. Table 3 summarizes the main themes and subthemes of emotional competency based on Malaysian parents' and teachers' perspectives.

**Table 2:** Cultural conceptualization of social competency based on Malaysian parents' and teachers' perspectives.

**Social competency**			
	**Theme 1:** preserving interpersonal relationships	**Theme 2:** utilizing intrapersonal skills	**Theme 3:** communicating effectively
CASEL subskills	**Sub-themes: -** (i) Having empathy ii) Being socially engaged iii) Establishing healthy social boundaries	**Sub-themes: -** i) Employing cognitive flexibility	**Sub-themes: -** i) Conveying appropriate content
Subskills related to Asian values		ii) Containing emotional dissatisfaction	ii) Embracing Asian communication etiquette

**Table 3:** Cultural conceptualization of emotional competency based on Malaysian parents' and teachers' perspectives.

**Emotional competency**				
CASEL subskills	**Theme 1:** practicing self-regulation **Sub-themes: -** i) Emotional awareness ii) Pause behavior iii) Emotional control	**Theme 2:** demonstrating help-seeking behavior		
Subskills related to Asian values			**Theme 3:** upholding altruism	**Theme 4:** maintaining cultural display rules

### 3.1. Social competency

#### 3.1.1. CASEL subskill

##### 3.1.1.1. Preserving interpersonal relationships

The ability of adolescents to establish and maintain relationships with others is an indicator of their social competence. Parents and teachers highlighted some crucial practices in preserving relationships, including both that enhance connection and limiting it, suggesting that balance in a relationship is essential to attain social competence. Practices that boost interpersonal relationships are demonstrating empathy and being socially engaged, whereas practice that limits particular social influence is maintaining healthy social boundaries.

###### 3.1.1.1.1. Having empathy

Empathy is one of the fundamental skills being taught in the established SEL practices, which lies under the domain of social awareness in the CASEL framework. There is even an SEL curriculum focusing on empathy as the main competency. Malaysian parents and teachers also recognize the importance of empathy as an essential quality of social competence. Participants described two types of empathy aligned with Goleman's remark of cognitive empathy and compassionate empathy (42). Practicing empathy improves adolescents' cognitive thinking in understanding the social world by applying perspective-taking:

“*So basically, about social competence, yeah, I think it lies in our children's ability to see the external world from their own perspectives and also able to appreciate others' perspectives as well”* (Parent #7).

From the participants' description, empathy does not just improve their social cognition; it also refines the adolescents' emotions and attitudes toward other people, which reflect compassionate empathy:

“*When our children have empathy, they will naturally greet other people from any walk of life and help others without needing us to push them much. They will naturally feel that they want to help and contribute”* (Parent #12).

A teacher also mentioned that showing empathy would enhance bonding and connection in relationships which builds up their social competence:

“*Yes, empathy.. Empathy will make you closer. The relationship will be closer. You can connect well with other people”* (Teacher #4).

###### 3.1.1.1.2. Being socially engaged

The ability to initiate and develop social engagement with other people is also one of the qualities that reflect social competence. This ability has been mentioned as the loss of common sense due to the advancement of technology. Hence, developing social engagement is emphasized as a competency that needs to be taught to Malaysian adolescents. Adolescents who put the effort to be socially engaged by interacting with other people and being physically present are considered to have high social competence. Developing positive relationships through social engagement falls under the relationship skills in the CASEL domain. Teachers reported that adolescents' social competency could be seen in their engagement in social environments like the classroom by responding to the learning experiences:

“*They are able to participate actively, okay.. They are verbal, they are active… they are not quiet in the class. They manage to contribute actively to the class discussion… The attitude that shows their presence in class”* (Teacher #10).

Participants also mentioned that social competency is declining among adolescents nowadays due to the increased usage of gadgets and technology. Adolescents' behavior of being quiet was attributed to the use of gadgets and perceived as a lack of effort to engage with other people:

“*Adolescents nowadays are very quiet, even in school. They are in their own world. Maybe with advances in technology. Even though I'm teaching the best class, the first class, I notice they are very quiet in class. They won't speak anything unless you push them and ask questions”* (Teacher #8).

“*I think adolescents nowadays are lacking in their social competency because they are very.. err very engrossed in gadgets. They don't mind not having real friends. They don't know how to talk to people and choose to be quiet most of the time in social situations”* (Teacher #3).

Moreover, parents noted that reduced face-to-face interaction due to the pandemic is a concern since adolescents are no longer used to engaging with significant others, which dampens family bonding:

“*I think it is a common problem in most families nowadays that their children are like.. away. I mean with all the gadgets, they are having less bonding time with parents.. like a face-to-face conversation”* (Parent #5).

“*We need to resolve the problems of how to like.. making our children have back the normal face-to-face communication skills like before (the pandemic). So, parents play an important role in this”* (Parent #1).

###### 3.1.1.1.3. Establishing healthy social boundaries

Social competency is also perceived as the ability to assert healthy boundaries in interpersonal relationships. Parents and teachers emphasized that establishing social boundaries indicates that adolescents are aware of the acceptable and unacceptable forms of social interactions and influences. This subskill falls under the domain of responsible decision-making in the CASEL framework since it involves making constructive choices about personal goals and social interactions in a specific context. A form of healthy social boundary is the ability to recognize toxic relationships in which adolescents may be misunderstood or manipulated. This boundary is essential to ensure emotional health and stability:

“*What is important is that they know that it is not always their fault not necessarily because of them. Sometimes other people really have problems with their own self. They need to know where the boundary is. We need to teach our children about boundaries so that they don't feel that they are the ones who are always wrong. Like.. what is wrong with me? Am I problematic?”* (Parent #10).

Another form of healthy social boundary reported by the participants is the ability of adolescents to filter negative social influences from peers and social media usage. This again involves the capacity to make constructive choices on internalizing the good and the bad influences. By assessing and asserting this boundary, adolescents are able to inhibit unhealthy and harmful social influences:

“*To me.. the adolescence phase is very fragile. So, if they mingle with the right crowd, they will be good. But if they mingle with the wrong crowd, it may give a bad influence on them”* (Teacher #5).

“*Sometimes, when they view their social media like TikTok or Facebook, they themselves need to be able to evaluate what is good and what is bad in dealing with the social media influence. That is an important part of being socially competent”* (Parent #1).

Our finding on establishing social boundaries has been surprising, considering Asian culture emphasizes social harmony over individual autonomy. In addition, parents and teachers also claimed that maintaining healthy social boundaries may sometimes undermine social hierarchy. Although Asian culture emphasizes respecting older and authoritative people like parents and teachers, adolescents are still encouraged to assert boundaries they deem healthy and helpful:

“*Respect.. yes, they need to respect. But if the teacher did something wrong, I say.. they need to know where the red flag is”* (Parent #6).

#### 3.1.2. Subskills related to Asian values

##### 3.1.2.1. Utilizing intrapersonal skills

Malaysian parents and teachers described social competency as the ability to control inner processes and internal attitudes to resolve external social circumstances. These intrapersonal skills are demonstrated by practicing cognitive flexibility and containing emotional dissatisfaction in dealing with social situations. Malaysian parents and teachers identified intrapersonal skills as a key to attaining interpersonal (social) competence, suggesting that SEC encompasses an interrelated set of skills.

###### 3.1.2.1.1. Employing cognitive flexibility

An intrapersonal skill that helps in navigating interpersonal relationships is cognitive flexibility. Cognitive flexibility allows for flexible thinking in shifting internal attention, adjusting the cognitive content, and switching behavioral responses to correspond to different social situations and tasks. The utilization of cognitive flexibility in adjusting to the social world reflects the domain of social awareness in the CASEL framework. One of the manifestations of cognitive flexibility is the ability to adapt to new social environments.

“*The way students adapt themselves to social environments, to me, is regarded as social competency. Whether in a family situation, in a classroom with teachers or in any other social circumstances... they know how to suit themselves”* (Parent #10).

This quality is endorsed by teachers at school: “*so we know students who have lower social competency, will be having a hard time to adapt with a new environment. He will need to take some time. Like in school, he will only greet his friends after recess time”* (Teacher #6*)*, and in contrast: “*students who are better at social skills are those who are able to adjust to both online and face-to-face classroom situations” (Teacher #2)*.

Employing cognitive flexibility would not only assist in navigating new social environments but also new people, which is why it is an essential component of social competency:

“*Okay, to me social competence means umm being able to meet with people, old or new. Being able to, you know… handle different kinds of people in their surroundings and mixing around is very important”* (Parent #11).

Cognitive flexibility also encompasses the ability to shift internal attention, which allows individuals to make efficient choices in life. A parent described that socially competent adolescents understand the concept of locus of control in handling social conflicts or dilemmas. This involves focusing only on things that are controllable and not dwelling on the uncontrollable circumstances: “*Sometimes they need to realize that they do not have control over everything”* (Parent #5).

###### 3.1.2.1.2. Containing emotional dissatisfaction

Containing emotional dissatisfaction is an aspect of social competence that involves the ability to suppress emotional experiences that are conflicted to prioritize social harmony in Asian culture. Parents and teachers emphasized that conflict is unavoidable when dealing with social situations. One of the practices in conflict management regarded as socially competent is to manage own emotional dissatisfaction rather than confronting it. This quality signifies selflessness by cherishing social harmony over individual ego. This adaptive skill in Asian culture is a component of experiential suppression that refers to direct attempts in suppressing the subjective emotional experience (43). This finding extended the conceptualization of the relationship skill domain in the CASEL framework:

“*So, every time there is a conflict, they need to think of ways to settle it. It is between settling it with the person involved or settling it by managing their own emotion discontent when facing the conflict. Dealing with own emotion is easier and safer.” (Parent #8)*.

“*In situations involving other people where they can contain their anger or stress, this indicates their quality of social competence”* (Teacher #1).

“*When they can control their emotion well, they will be more likable*” (Parent #3).

Containing emotional dissatisfaction is described as a crucial skill in becoming socially competent and accepted. Another social circumstance that would benefit from one's ability to contain emotional dissatisfaction is to celebrate differences:

“*If others' opinions are not the same as theirs, how they react emotionally in a way that it won't hurt other people is a kind of social competence too, you know”* (Parent #7).

##### 3.1.2.2. Communicating effectively

Malaysian parents and teachers highlighted that social competence is highly influenced by the ability to communicate effectively with manners. Two qualities of communication expected from adolescents are the ability to convey appropriate content and to embrace Asian communication etiquette. While ensuring appropriate content facilitates adolescents on what is considered effective communication, embracing Asian etiquette facilitates how to communicate respectfully in Malaysian culture.

###### 3.1.2.2.1. Coveying appropriate content

The quality of the message conveyed is perceived as an integral part of effective communication. The ability of adolescents to choose appropriate words in communicating with other people, especially those who are older and authoritative, indicates their social competence. This quality falls under the domain of relationship skills in the CASEL framework since effective communication is crucial for meaningful social interaction. Parents and teachers reported that socially competent adolescents are those who are able to restrict themselves from using bad language or inappropriate youth jargon even though it has been the communicating style of other peers around them:

“*Good communication means they mind the choice of words that they use. Adolescents must have their own style of language... you know, the adolescents' jargon. But they know that they should not use it with parents and teachers”* (Parent #5).

“*Some students came to school and talked using bad language. Maybe the students are used to it at home. Certain words may sound rude like they always say, 'so what, teacher?', and this somehow shows their level of social competence”* (Teacher #1).

Teachers described that another aspect of conveying appropriate content includes delivering meaningful messages and conveying their needs across. This basic communication skill was lost due to the prolonged period of social quarantine throughout the pandemic:

“*Socially competent students would portray good communication skills. I think everyone can talk. But the… the ability to express one thing in a way that delivers actual meaning seems to be hard for our students nowadays, you know, after the pandemic and everything... I mean, the meaning needs to be there. And we know actually what the objectives is… clear objectives”* (Teacher #10).

“*Students nowadays have a lot of problems. But they can't manage to, like… put it in proper words, especially when in a face-to-face conversation. They can't express their needs or the help that they are seeking in words. This has something to do with their social competency too, you know”* (Teacher #7).

###### 3.1.2.2.2. Embracing Asian communication etiquette

Participants described some etiquettes in communication regarded as the foundation of adolescents' social competence. These etiquettes include voice intonation, sentence segmentation, and eye contact closely matched with Asian values and communication styles. The etiquette emphasized respectful gestures when communicating with older and authoritative people. This quality contributes to a unique conceptualization of subskill under the relationship skill domain of the CASEL framework. Example of Asian communication etiquette includes using a soft voice intonation when conversing with older people and maintaining eye contact to show attentiveness:

“*Social competence is when our children can incorporate ethics and manners in communication... the voice intonation that they use, how many segmentations and pause depending on whom they speak to”* (Parent #12).

“*Yes, the intonation. The way.. the eye contact, students don't make eye contact. Even when the teacher is asking questions, they don't look at the teacher to answer. I feel that is quite rude. Yeah? I don't know… sometimes I feel disturbed”* (Teacher #4).

“*Aaa language intonation. Not just intonation of verbal communication, but the use of right body language needs to be taken into consideration too”* (Teacher #6).

A parent also claimed that adolescents are expected to uphold their etiquette when communicating with older people despite the closeness of relationships or spontaneous conversations:

“*I always say this to my children, since we are close… you can talk to me like a friend, but at the same time, you have to remember that I am your mother. Okay, you can talk like a friend to your teacher but remember at the same time that she or he is your teacher”* (Parent #3).

### 3.2. Emotional competency

#### 3.2.1. CASEL subskills

##### 3.2.1.1. Practicing self-regulation

Emotional competency is viewed as the ability to apply self-regulation practices. Skills such as emotional awareness, pause behavior, and emotional control facilitate adolescents' self-regulation and become indicators of their emotional competence. These skills are also endorsed by the CASEL framework under the domains of self-awareness and self-management.

###### 3.2.1.1.1. Emotional awareness

Participants mentioned that the ability to identify emotions present in oneself and others is the fundamental step in responding to and regulating emotions effectively. This skill falls under the domain of self-awareness in the CASEL competencies:

“*They need to recognize their emotion to identify the reason their emotions are disturbed”* (Teacher #9).

“*Emotional competency is when our children can recognize emotions that they experienced and then take action to regulate the emotions”* (Parent #11).

Another aspect of emotional awareness is acknowledging the emotions experienced. Participants cited that “*every emotion needs to be acknowledged”* (Parent #2) and “*acknowledging emotions helps them to know when to manage certain emotions”* (Teacher #5).

###### 3.2.1.1.2. Pause behavior

Several participants emphasized practicing pause behavior as a sign of emotional competence. Pause is perceived as a practical approach to managing emotions and behaviors by allowing clarity to choose desired actions rather than allowing emotions to drive impulsive behaviors. Therefore, this skill falls under the domain of self-management in the CASEL competencies. A teacher quoted that emotional competency involves not being impulsive or reactive in dealing with emotional conflicts:

“*For example, when they are stressed or too mad, they can choose not to react at that time or just pause for a while and take their me time to think and respond later”* (Teacher #9).

According to some parents, social competence involves the ability of adolescents to seek their own space and calm down before addressing intense emotions or problems:

“*They know to take space to calm down and cool down first”* (Parent #10).

“*Space… they know how to cool down and whatever they are feeling, they can express after that”* (Parent #9).

###### 3.2.1.1.3. Emotional control

Emotional control is another self-regulation practice that indicates the emotional competency of adolescents. It falls under the domain of self-management in the CASEL competencies. Teachers mentioned that students with good emotional competency are able to manage the extent to which emotions can influence their thoughts and behaviors. Emotional control refers to the ability of adolescents to control the domination of emotions over their lives:

“*What I understand about emotional competency is that they are able to handle emotional situations, they can control themselves and… they do not be carried away when they are emotionally affected, you know? They are very wise when handling it”* (Teacher #6).

“*Emotional competency is when someone tries to control their emotion so it will not go far. The emotion is just there and stops there. They know how to control their emotion so that it will not affect them negatively”* (Teacher #3).

Another parent cited that emotional control entails the ability to alter the intensity of emotional experience and reactions:

“*… to control their emotions so they will not be hyper or too sad or too stressed especially when it comes to facing the examination”* (Parent #4).

Adolescents exhibit emotional competency when they are able to control their emotions according to their knowledge, spirituality, and cultural aspects:

“*Whatever they are feeling, they can still control themselves… you know, based on their knowledge, experience and exposure toward spiritual and cultural aspects”* (Parent #2).

##### 3.2.1.2. Demonstrating help-seeking behavior

Malaysian parents and teachers pointed out that emotional competency encompasses the ability of adolescents to demonstrate help-seeking behavior. This practice involves reaching out and opening up to seek emotional support and help, which are referred to as interpersonal ER in the literature (44). Demonstrating help-seeking is viewed as a sign of emotional competence since it involves not just acknowledgment of emotional problems but also appraisal of the problems faced. This skill falls under the domain of self-management in the CASEL framework. Emotionally competent adolescents are aware of their emotional condition and the degree of severity that warrants intervention:

“*There is an extent to which they feel like 'oh, I need help, I need to seek help'… If they can identify that, this indicates their emotional competence”* (Teacher #8).

“*They know that sometimes they need to seek help. They are aware of which emotional conditions need intervention. Because we do not want things to get more severe and chronic, right?”* (Parent #1).

Adolescents with high emotional competency are also capable of identifying maladaptive-prolonged emotional problems and choose to seek help:

“*They are aware of when they need to seek help… the duration of their emotional problems. They know when they could no longer solve their emotional problems by themselves and choose to seek help”* (Parent #4).

However, from the participants' descriptions, help-seeking behavior primarily focuses on the familial and community approach. Parents and teachers value adolescents' ability to reach out and share their emotional problems with adults in the family or community. Unfortunately, there was no professional help mentioned by the parents and teachers, which supports studies reporting delays in professional help-seeking for mental health disorders among Asian due to first-line familial and community preferences (45–47). Peers were regarded as the least preferable individual to seek help to:

“*In managing emotional problems, the most ideal would be they try to share and seek help through… firstly through prayers. And then secondly share with their parents or teachers whom they trust, or they are closed with… some adolescents have trust issue with parents, they are closer with their teachers, so no problem at all to open up their emotional problems to the teachers. Next, maybe they can share with the extended family whom they believe could help them, and peers are the last option”* (Parent #9).

Parents and teachers were concerned about their children and students who may be struggling emotionally on their own. Without emotional support, emotional problems could escalate into more serious mental disorders. Hence, they genuinely wish to intervene and offer their help in addressing adolescents' emotional problems. This could only be achieved if the adolescents are able to demonstrate help-seeking behavior:

“*If they can open up to us or show us in a way that we can see clearly what their emotional problems are, then I will be able to you know umm… I'll be able to help the child, speak to the child, or get nearer to the child so that things will not get severe”* (Teacher #3).

#### 3.2.2. Subskills related to Asian values

##### 3.2.2.1. Upholding altruism

A distinctive finding of emotional competency that reflects Asian values is the ability to uphold altruism in dealing with emotional conflicts. Altruism is a moral principle concerning for happiness and benefit of other people. Even though altruism indicates more about prosocial behavior instead of emotional competency, according to Malaysian parents and teachers, upholding altruism reveals emotional competency since it involves the fundamental element of abstaining and forgoing one's emotions to prioritize societal harmony. It is an emotional competency manifested in interpersonal circumstances, suggesting that SEC encompasses an interrelated set of skills. This quality expands the conceptualization of subskill unique to Malaysian culture under CASEL's responsible decision-making domain since it involves making caring choices about personal behavior and social interactions. Upholding altruism serves as the intrinsic motivation and guiding principle in managing emotions which results in more positive and harmonious consequences for the people involved:

“*Emotional competency, as I understand, is when my children can think of ways to manage their emotions that will eventually bring positive action and be considered a win-win for everybody. They know how to balance between prioritizing their own emotion and situations that involve other people”* (Parent #11).

“*Students who can manage their emotional outlet and responses in a way that will not give a bad impact on other people”* (Teacher #7).

Several participants quoted that emotional competency is also manifested in adolescents' ability to abstain from personal egoism by empathizing with others in attending to interpersonal emotional conflicts:

“*Emotional competency is when the adolescent is not selfish in dealing with interpersonal emotional conflicts. They try to see problems from other people's perspectives as well… they know that they are not necessarily always right. Like…they can empathize with others”* (Parent #8).

##### 3.2.2.2. Maintaining cultural display rule

While upholding altruism serves as the intrinsic motivation in emotional management, maintaining cultural display rules imparts an external manifestation of the process. Cultural display rules are the social and cultural norms that influence the appropriate and acceptable expression of emotions. Adolescents who are able to perform culturally appropriate modifications in their emotional expression are considered emotionally competent. The main objective in maintaining appropriate display rules in Asian culture is to respect older and authoritative people and preserve relationships and harmony during emotional conflicts or intense situations. Hence, it belongs to the domain of relationship skills of the CASEL competencies since it facilitates harmonious relationships in Malaysian culture.

This adaptive skill in Asian culture is a component of expressive suppression in ER (29, 43). Malaysian parents and teachers described cultural display rules expected in adolescents involve minimizing and masking some emotional expression. For example, since respecting older and authoritative figures is significant in Asian values, adolescents are supposed to control intense and conflicting emotions by minimizing their emotional expression to uphold their respect:

“*Emotional competency is about knowing the right way to express emotions. To show that they are mad, but at the same time, still want to respect the person. For example, my child, when he is mad, but he still shows that he respects me as his mother”* (Parent #3).

Another form of emotional display rule highlighted by the participants is the ability of adolescents to mask certain emotions in challenging circumstances by expressing their calmness and patience during social conflicts:

“*When the students can maintain their calmness and patience in front of other people, this shows how well they can manage their emotions”* (Teacher #8).

“*Emotional competency in adolescents can be seen when they are able to be patient. They can show calmness in dealing with emotional problems”* (Parent #6).

Since Asian culture appreciates social harmony, participants denoted that sustaining positive affect and masking negative affect are also part of the emotional display rule that indicates adolescents' emotional competency:

“*Part of emotional competence is the ability to show affection to other people, smile to their peers, to their teachers and so on”* (Teacher #2).

“*I've seen students wow… very cheerful, very jovial, very happy, very active in class, but they are from problematic family”* (Teacher #7).

## 4. Discussion

This formative study explored Malaysian parents' and teachers' cultural conceptualization of adolescent SECs. Malaysian parents' and teachers' beliefs and understanding of what constitutes crucial SEC for adolescents were studied as part of a systematic cultural adaptation process from the ground up before implementing SEL in Malaysian education. Our findings suggested that the conceptualization of SEC in the Malaysian context is aligned with the established CASEL framework. Malaysian parents and teachers denoted similar conceptualization of subskills under the following five domains of CASEL competencies: self-awareness, self-management, social awareness, relationship skill, and responsible decision-making (Table 4). This implied the feasibility of SEL curriculums and modules based on the CASEL framework to be implemented in Malaysia.

**Table 4:** Themes and subthemes of SEC according to CASEL domains and subskills.

**CASEL domains of competencies**	**CASEL subskills**	**Subskills related to Asian values**
**Self-awareness**: the abilities to understand one's own emotions, thoughts, and values and how they influence behavior across contexts	- Practicing emotional awareness	
**Self-managements**: the abilities to manage one's emotions, thoughts, and behaviors effectively in different situations and to achieve goals and aspirations	- Practicing pause behavior - Practicing emotional control - Demonstrating help-seeking behavior	
**Social awareness**: the abilities to understand the perspectives of and empathize with others, including those from diverse backgrounds, cultures, & contexts	- Employing cognitive flexibility Having empathy	
**Relationship skills**: the abilities to establish and maintain healthy and supportive relationships and to effectively navigate settings with diverse individuals and groups.	- Being socially engaged Conveying appropriate message	- Containing emotional dissatisfaction - Embracing Asian communication etiquette - Maintaining cultural display rules
**Responsible decision-making**: the abilities to make caring and constructive choices about personal behavior and social interactions across diverse situations	- Establishing healthy social boundaries	- Upholding altruism

In addition to covering all domains of the CASEL competencies, our themes and subthemes also extended the conceptualization of subskills under some CASEL domains according to Asian cultural values pertaining to social harmony and social hierarchy. Our findings revealed that Malaysian parents and teachers endorsed a self-concept defined by social embeddedness and interdependence with others which are core features of collectivist cultural orientation (48, 49). Malaysia, along with other Southeast Asian countries, has been known as a collectivist country, and this study adds to the evidence supporting this claim in the case of Malaysia. Two CASEL domains that highly reflected the Malaysian collectivist culture are relationship skills and responsible decision-making (Table 4). This is where the cultural adaptation process needs to be employed to ensure a culturally sensitive SEL intervention for Malaysian education.

Social and emotional competencies that relate to Asian collectivist values are discussed further by first highlighting suppression as an adaptive ER strategy for adolescents in Malaysia. Second, we offered a potential alternative ER strategy as a point to intervene through effective and respectful communication that honors Asian etiquette. Third, we suggested a culturally sensitive intervention known as expressive writing exercise to accompany the habitual use of emotional suppression in an emotional restraint culture like Malaysia. Finally, we outlined some strengths and limitations of the current study and provided recommendations for future directions.

### 4.1. Suppression: An adaptive emotion regulation strategy in a collectivist culture

Our study supported previous findings noting suppression as an adaptive ER strategy in a collectivist culture (24–29). Malaysian parents and teachers highlighted the importance of upholding altruism that serves as a principle of preserving social harmony and achieving collective goals. This principle represents the domain of responsible decision-making in the CASEL competencies since being responsible in making decisions in the Malaysian collectivist context involves upholding the altruism principle. Malaysian parents and teachers emphasized some approaches in line with the altruism principle under the relationship skill domain, which are containing emotional dissatisfaction, maintaining cultural display rules, and embracing Asian communication etiquette.

**Figure 1:**
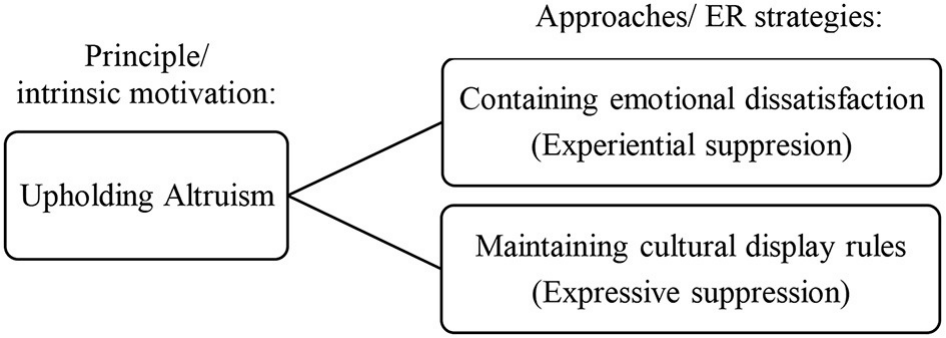
Potential drive toward adaptive suppression in Malaysian collectivist culture.

From here, we recognized patterns of adaptive suppression in collectivist culture deemed as crucial competencies for adolescents in Malaysia. For instance, containing emotional dissatisfaction in resolving interpersonal conflicts implies the use of experiential suppression in which the subjective emotional experience like anger or disappointment is suppressed there and then to preserve harmony. In addition, the ability of adolescents to maintain cultural display rules as an indicator of emotional competence denotes the use of expressive suppression in which the outward display of emotions is inhibited and concealed to show respect to someone older and authoritative. We postulated that the intrinsic motivation for selecting suppression as a preferred ER strategy in Malaysian collectivist culture comes back to altruism as a core principle. This postulation is worth to be studied further in future research to understand the underlying factor, intrinsic or extrinsic motivations toward emotional suppression in Asian context. Figure 1 summarizes our conceptual postulation regarding the drive of upholding altruism as a potential intrinsic motivation toward choosing suppression as an adaptive ER strategy.

Our findings that expanded the subskills of the CASEL framework according to Asian collectivist values are parallel with the adapted SEL framework used in Singapore. In addition to the five domains of CASEL competencies, Singapore's SEL framework posits Asian core values as central guidance in utilizing the SEL competencies. This is to emphasize that the implementation and teaching of SECs should be rooted and grounded in cultural values (30). The six core values introduced in Singapore's SEL framework include respect, responsibility, integrity, care, resilience, and harmony, which supports their community's collectivist cultural orientation (30, 31).

### 4.2. Effective and respectful communication: A point to intervene

Although our findings implied that emotional suppression is adaptive and favorable to Malaysian collectivist culture, Malaysian parents and teachers also highlighted the importance of help-seeking behavior, indicating an alternative interpersonal ER strategy. Malaysian parents and teachers highly valued the ability of adolescents to reach out and share their problems and emotional concerns. This suggested that the emotional health of adolescents matters and is prioritized. In addition, our findings also reported that there are instances whereby the importance of emotional health surpasses the demands of collectivist culture to preserve harmony. Malaysian adolescents are encouraged by their parents and teachers to pursue autonomy in establishing social boundaries if interpersonal relationship disrupts their emotional health and stability. This suggested that suppression is not the only potential ER pathway regarded as culturally sensitive in Malaysia.

**Figure 2:**
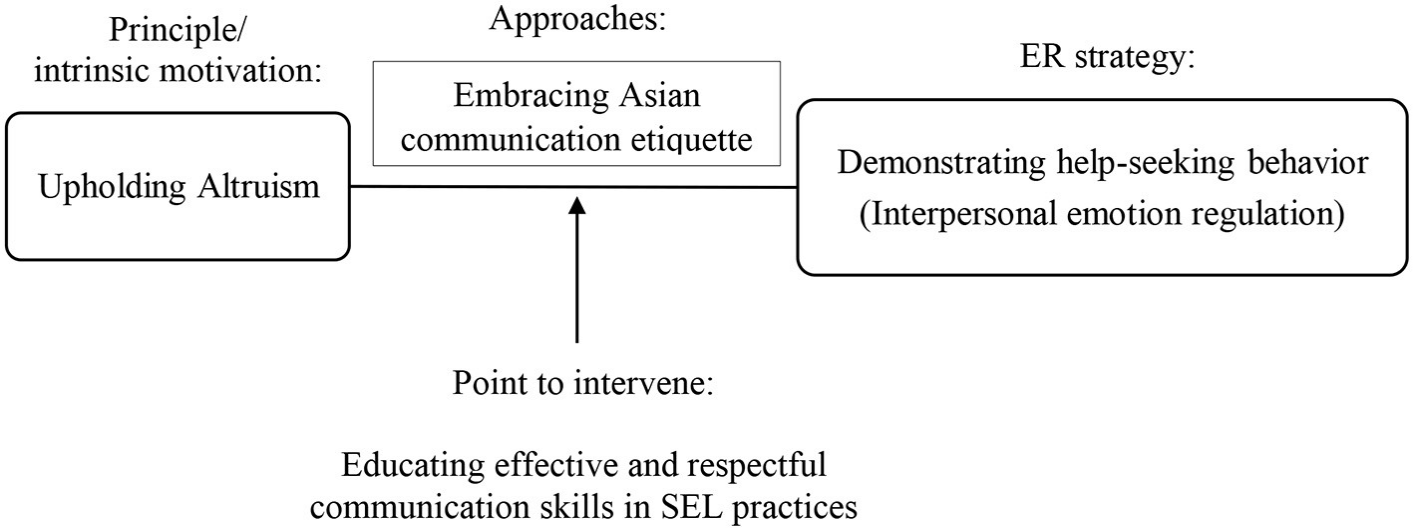
Potential alternative emotion regulation pathway for Malaysian collectivist culture.

Among the approaches to upholding the principle of altruism other than experiential and expressive suppression is the alternative strategy of communicating respectfully with Asian etiquette (Figure 2). In the Malaysian context, respectful communication following Asian etiquette is key for adolescents to convey their needs to adults around them. We would like to suggest this alternative pathway as a point to intervene in culturally adapting SEL intervention for Malaysian adolescents. A primary goal of SEL in Malaysia should be devoted to teaching students to communicate their social and emotional needs effectively while respecting Asian communication etiquette. SEL modules focusing on educating effective communication have already been implemented in other countries (50, 51). In Malaysia, lessons on communication skills need to be adapted with the appropriate communication etiquette that honors collectivist values. Fostering effective and respectful verbal communication between adolescents and adults in Malaysia would facilitate a better understanding of adolescents' social and emotional needs and difficulties. This will potentially promote interpersonal ER strategy among Malaysian adolescents through help-seeking behavior as valued and desired by Malaysian parents and teachers (Figure 2).

Second, due to the habitual use of emotional suppression in the Malaysian collectivist culture, we proposed the expressive writing intervention as one of the culturally sensitive SEL practices in Malaysia to improve adolescents' self-awareness and self-management. This practice of emotional disclosure through writing has been found to be effective, particularly among adolescents in improving emotional awareness and coping strategy (52, 53). A growing body of evidence suggests that expressive writing exercises promote wellbeing by improving perceptions regarding emotions and self-concept (54). Through expressive writing, one can overcome emotional avoidance and suppression through meaning-making that utilizes cognitive, affective, and motivational elements (55). According to the “affective adaptation theory,” negative emotional reactions to events decrease with a deeper understanding of them, which makes expressive writing a key component of self-regulation (56, 57). This technique may offer a unique opportunity for Malaysian adolescents to express and process their emotions without worrying about relational social consequences or relationship repercussions (58). Expressive writing is a brief psychological intervention that bypasses the cognitive demand of respectful verbal communication between Malaysian adolescents and adults. This is not to say that expressive writing may replace the need for effective communication; however, it is an easier and quicker adaptive lesson to improve adolescents' wellbeing. Educating expressive writing intervention together with effective and respectful communication skills would enhance the cultural fit of SEL intervention in Malaysia.

### 4.3. Strength and limitations

A key strength of this current study is that it fulfills the niche in global SEL adaptation research by providing empirical data specific to a cultural group in the Southeast Asian region. This study provides a nuanced understanding of adaptive and normative ER strategies of experiential and expressive suppressions in a collectivist country like Malaysia. Its qualitative nature allows deeper exploration into the underlying and intrinsic motivation of emotional suppression which is to uphold the altruism principle. It also offers a potential alternative ER strategy suitable for Malaysian adolescents that embrace the respectful Asian style of communicating.

One of the limitations of this study pertains to the heterogeneity of cultures in Malaysia. Even though we did incorporate participants from three different races, it may not be adequate in representing Malaysian cultural perspectives as a whole due to the richness of other sub-ethnicities. Second, the homogeneous nature of most focus groups that consist of only Malay participants may not result in an optimum dynamic that provides in-depth understanding of cultural conceptualization in the Malaysian context. The safe homogeneous environment of the Malay groups may breed single-mindedness that could emerge into one-sided data (59). Third, this study was limited to exploring the perspectives of parents and teachers who deal with adolescents in concluding the Malaysian conceptualization of adolescents' SEC. As the saying goes, “*It takes a village to raise a child*”; therefore, opinions from more stakeholders such as counselors, parenting experts, and other folks who are not necessarily parents or teachers to an adolescent should also be taken into consideration.

## 5. Conclusion

By studying Malaysian parents' and teachers' cultural conceptualization of adolescent SEC, this formative study informed the feasibility of SEL modules developed based on the CASEL framework to be implemented in Malaysia. In addition, we suggested two key lessons to enhance the cultural sensitivity of SEL modules in Malaysia: effective, respectful communication and expressive writing exercises.

## 6. Recommendations

Future directions following this formative study would be used to triangulate data from this cultural study with the adolescent study to develop a culturally sensitive SEL framework for Malaysian adolescents. The parallel effort needs to be channeled to address the habitual use of suppression as the preferred ER strategy in Malaysia. The hallmark features of SEL are more than just molding adolescents to behave in a certain acceptable way. It actually nurtures character strength, awareness of self and others, healthy coping skills, and many more. Hence, groundwork in the process of adapting SEL in Malaysia may include educating or raising awareness among parents and the community on the importance of healthy social and emotional development for children and adolescents. In addition to that, efforts in improving parents' and educators' SEC should be incorporated to facilitate the best delivery of SEL intervention in Malaysian education. In addition, due to the unique collectivist orientation in Malaysia, culturally sensitive assessment tools should be explored to measure the level of SEC among Malaysian children and adolescents. Future studies could be useful to explore the benefit of Asian collectivist values in supporting SEL. The significant strength of families and communities in a collectivist culture should be integrated with future SEL adaptation.

## Data availability statement

The original contributions presented in the study are included in the article/supplementary material, further inquiries can be directed to the corresponding author/s.

## Ethics statement

The studies involving human participants were reviewed and approved by Research Ethics Committee of the National University of Malaysia. The patients/participants provided their written informed consent to participate in this study.

## Author contributions

Conceptualization of research: MM, LC, HM, AA, and NA. Data collection: NA and SA. Analysis of data: NA and ST. Writing: NA. Review of manuscript: MM, LC, and ST. All authors contributed to the article and approved the submitted version.
